# Effect of Pandemic on the Clinical Status of Patients Admitted to Hospital for Diabetic Foot: A Retrospective Study

**DOI:** 10.3390/jcm14145067

**Published:** 2025-07-17

**Authors:** Seda Pehlivan, Hülya Ek, Semure Zengi, Suzan Adalı, Özen Öz Gül, Soner Cander, Canan Ersoy, Erdinç Ertürk

**Affiliations:** 1 Department of Nursing, Health Science Faculty, Bursa Uludag University, Bursa 16059, Turkey; 2 Bursa Uludag University Hospital, Bursa 16059, Turkey; hulyaek22@gmail.com (H.E.); semurezengi@uludag.edu.tr (S.Z.); suzanadali@hotmail.com (S.A.); 3 Department of Endocrinology and Metabolism, Faculty of Medicine, Bursa Uludag University, Bursa 16059, Turkey; ozenoz@uludag.edu.tr (Ö.Ö.G.); scander@uludag.edu.tr (S.C.); ecanan@uludag.edu.tr (C.E.); ererturk@uludag.edu.tr (E.E.)

**Keywords:** diabetes mellitus, diabetic foot, pandemic

## Abstract

**Background/Objectives**: Diabetic foot (DF) is among the leading causes of diabetes-related disability. It is important to maintain regular follow-up and patient education in the prevention and treatment of DF ulcers. In extraordinary situations such as a pandemic, there are disruptions in regular clinical follow-up and patient education, and the effects of this disruption need to be investigated. The aim of this study was to investigate the impact of the pandemic on the clinical condition of patients hospitalised for DF. **Methods:** Patients were divided into two groups according to the date of admission to the clinic: the pre-pandemic (1 January 2019–11 March 2020) and the pandemic period (12 March 2020–1 June 2021). Comparisons were made between the two groups in terms of DF data and clinical parameters. Data were analysed with SPSS using chi-square, Student’s *t*-test and Mann–Whitney U analysis. **Results:** As a result of the screening, data from 125 DF patients (45 pre-pandemic and 80 pandemic) were collected. The DF stage, according to the Wagner classification, was significantly more advanced in patients during the pandemic period (*p* = 0.015). However, the time between the onset of symptoms and hospitalisation was longer for patients during the pandemic period (*p* = 0.035). When analysing treatment outcomes, the rate of wound healing was found to be lower (62.2% vs. 30%), and the rate of transtibial amputation was higher (11.2% vs. 20%) during the pandemic period (*p* = 0.002). **Conclusions:** This study found that the number of patients hospitalised for DF increased during the pandemic period, as did the severity of the wound, length of admission and radical treatment interventions.

## 1. Introduction

The COVİD-19 disease, which emerged in Wuhan, China, at the end of 2019, quickly spread to other countries and affected the whole world. This new disease caused an unexpected change in the economy and society around the world [1]. The first case in our country was reported on 11 March 2020, and the World Health Organisation (WHO) declared a pandemic on the same day [2]. Some precautions were implemented in healthcare systems to alleviate the burden caused by the new disease. To make room for pandemic patients, the number of inpatients and outpatient appointments was reduced. As a result, people with milder problems due to their chronic illnesses were hospitalised less frequently [3]. On the other hand, it is known that diabetes mellitus (DM), which is considered a global epidemic, has continued to increase rapidly in our country and in the world. During the time of the double pandemic, when two pandemics occurred side by side, the poor COVID-19 prognosis in DM patients was emphasised due to the large number of patients affected [4]. As people with diabetes represent a vulnerable population with a high risk of COVID-19 mortality, they were also included in the WHO’s definition of “high-risk and vulnerable groups” [5]. To reduce the risk of exposure to COVID-19 during the pandemic, it was therefore recommended to avoid diabetes-related hospitalisations unless absolutely necessary [3]. However, health authorities repeatedly emphasised that the elderly and people with chronic illnesses should stay at home and isolate themselves. Due to the quarantines/restrictions imposed during the pandemic, the number of follow-up visits and early doctor visits dropped to less than half, and consultations were limited to severe cases and emergencies [1].

Diabetic foot (DF) ulcers are one of the most common causes of hospitalisation in people with diabetes and affect one-third of all people with diabetes [3]. DF, one of the leading causes of disability due to diabetes, is also the leading cause of amputations independent of diabetes. DF defines ulceration, infection or progressive disease of the foot in people with diabetes [6]. Risk factors for DF in patients with diabetes include microvascular complications, distal peripheral neuropathy, peripheral arterial disease, recurrent trauma, previous ulcers and previous amputations [7,8]. The two most significant risks in the pathophysiology of DF are primarily distal peripheral neuropathy and peripheral vascular disease [7]. Metabolic, cardiovascular, and inflammatory changes leading to nerve injury, particularly in the distal extremities, play a role in the pathophysiology of diabetic neuropathy. These changes cause numbness, pain and ultimately sensory loss throughout the sensory nerves, particularly in the feet. The sensory sural nerve is particularly vulnerable to diabetic damage, making it a significant factor in diabetic foot deterioration [9]. Patients who experience sensory loss due to diabetic neuropathy cannot adequately assess their risk of DF and may fail to maintain preventive foot self-care practises [7,10]. It is easier to prevent DF wounds than to treat and manage their consequences. Therefore, patients should be informed about the importance of preventive foot care practises, and the practise status should be monitored [11]. However, early detection, diagnosis and treatment play a key role in reducing DF-related morbidity and improving clinical outcomes. However, DF patients had difficulty accessing outpatient services during the COVID-19 pandemic [10].

DF is a clinical condition that requires intensive treatment, takes months to heal and carries the risk of repeated hospitalisation and amputation. It is estimated that 20 million people worldwide suffer from DF, and 130 million people have a significant risk factor, resulting in approximately nine million hospitalisations and two million amputations per year [6]. During the COVID-19 pandemic, the interruption of regular check-ups for patients with diabetes led to a decrease in the assessment of risk factors for DF and the possibility of early treatment. In addition to patients’ fear of contracting the disease, hospital admissions were reported to be delayed due to patient restrictions imposed in hospitals [7]. It has been emphasised that this can lead to the progression of DF wounds and make treatment difficult. However, it has also been argued that the quarantine practise may have allowed patients to avoid walking, so that the DF ulcers healed [1]. Although there are initial studies in the literature, it has been reported that long-term comparative data and real data from low-/middle-income countries are needed [6,12]. Considering that data from middle-income countries, including our country, are limited, and that there are few comparative studies on DF specifically in the context of the pandemic, the data from our study will make an important contribution to the literature. Furthermore, the results of this study are considered important for better understanding the impact of the pandemic on the DF clinic and for highlighting the need to restructure clinical protocols. Accordingly, the aim of our study was to investigate the impact of the pandemic on the clinical status of patients hospitalised for DF.

## 2. Materials and Methods

Data were collected retrospectively using a data collection form. This study analysed the records of patients who were admitted to the endocrinology outpatient clinic for DF between 1 January 2019 and 1 June 2021 and treated as inpatients in the clinic. Patients were divided into two groups depending on the date of admission: the pre-pandemic period (1 January 2019–11 March 2020) and the pandemic period (12 March 2020–1 June 2021). Comparisons were made between the two groups in terms of DF data and clinical parameters.

### 2.1. Data Collection and Analysis

The data were collected by the researchers from the electronic file system over three months from 20 September 2021. As there was no DM/DF outpatient clinic in our hospital, a search was conducted using the keywords “diabetes” and “diabetic foot” among patients who had been registered in the endocrinology outpatient clinic and inpatient clinic between 1 January 2019 and 1 June 2021. The numerical data obtained through the search were also recorded by the researchers (Table 1). As a result of the search, data from 125 patients diagnosed with DF were obtained, and the relevant information from the electronic records of these patients was entered into the data collection form.

In the data collection form, sociodemographic characteristics of the patients (age, gender, marital status, place of residence, smoking and alcohol consumption), disease characteristics (duration of diabetes, treatment, follow-up status, history of DF, comorbidity and additional findings such as diabetic nephropathy) were recorded, A total of 20 questions were asked about metabolic values (fasting blood glucose-FBG, HbA1c, CRP, Erythrocyte sedimentation rate-ESR, leucocytes, Hg and creatinine) and DF characteristics (time between symptom onset and hospitalisation, Wagner classification, presence of osteomyelitis, treatment and treatment outcome).

For the metabolic data, the results of the tests performed at the time of hospitalisation were recorded. The assessment of the treatment outcome was based on the patient’s assessment notes at the first follow-up examination after admission.

### 2.2. Statistical Analysis

The data obtained were analysed using SPSS v28.0 (IBM Company, Chicago, IL, USA). The normal distribution of the data was analysed using Kolmogorov–Smirnov analysis. According to the results of the normality analysis, it was found that of the continuous variables, only age and haemoglobin level were normally distributed. Accordingly, the chi-square test, Student’s *t*-test and Mann–Whitney U-test were used to compare age and haemoglobin levels, as the other continuous variables did not have a normal distribution. A *p*-value of < 0.05 was considered significant.

### 2.3. Ethical Approval

The scientific research application for our study was approved by the Ministry of Health (2021-08-06-06T16_58_31). The required authorisation was obtained from the Ethics Committee for Clinical Research (date: 8 September 2021 and no.: 20 December 2021).

## 3. Results

In accordance with the data obtained in our study (Table 1), it was found that the number of DM patients hospitalised (*p* < 0.001) and hospitalisations decreased during the pandemic period, while the number of DF patients increased (*p* < 0.001). Of note, the number of patients hospitalised for DM increased to 90% during the pandemic (*p* < 0.001). Although the number of DF admissions increased during the pandemic period (*p* < 0.001), the number of hospital admissions decreased. For all DM patients who were hospitalised, the rate of hospital admissions due to DF increased during the pandemic period (*p* < 0.001).

It was determined that there was no difference in the sociodemographic characteristics of patients diagnosed with DF before and during the pandemic (*p* > 0.05) (Table 2).

When analysing the distribution of data on disease characteristics (Table 3), it was found that the duration of diabetes mellitus was longer in DF patients admitted during the pandemic period (*p* < 0.05). In the pre-pandemic period, patients with a history of DF were found to be in the majority, while during the pandemic period, the majority of patients had no history of DF (*p* < 0.05).

There was no difference between the periods in terms of follow-up status, comorbidities and findings (*p* > 0.05). The metabolic scores of patients diagnosed with DF were found to be similar before and during the pandemic period (*p* > 0.05) (Table 4).

The distribution of DF data by time period is shown in Table 5. During the pandemic period, the time from wound discovery to hospitalisation was found to be longer (*p* < 0.05). However, the proportion of patients admitted with stages 1 and 4 decreased during the pandemic period, while the proportion of patients with stages 2, 3 and 5 increased (*p* < 0.05). There was no difference between the periods in terms of the presence of osteomyelitis, hospitalisation status and duration of hospitalisation. In the treatments applied, it was observed that antibiotherapy was started in all patients, and blood glucose regulation and DF education were provided. However, there was no difference between the periods in terms of treatments administered in the hospital (*p* > 0.05). Among DF patients followed up during the pandemic period, the wound healing rate decreased from 62.2% to 30%, the rate of wound regression increased from 13.7% to 37.5%, and the rate of patients with transtibial amputation increased from 11.2% to 20% (*p* < 0.05).

## 4. Discussion

The pandemic period led to an increase in stressors such as quarantine, low physical activity, less contact with healthcare staff and a direct negative impact on patient health [13]. During the pandemic, precautions were taken in clinics, and the number of inpatients in clinics outside the pandemic period was reduced. However, most people with chronic conditions such as DM were not followed up regularly due to fear of contracting the virus or self-isolation precautions, leading to indirect effects of the pandemic. Pandemic measures have been shown to hinder blood glucose control and the treatment management of patients with diabetes who require regular follow-ups. Failure to maintain routine diabetes control has been reported to increase diabetes-related complications, including DF amputations and lower limb amputations [14]. The COVID-19 pandemic has been a new and difficult time for the whole world to cope with. Therefore, it is very important to evaluate the significance and impact of the pandemic period on patients with DM. In a study, it was found that the problems faced by patients with DM during the pandemic period were difficulties controlling blood sugar levels, adhering to diet and the inability to exercise regularly at home because they could not go out much. However, it was found that they wanted to be informed about how a person with diabetes would be affected by the pandemic (61.5%) and to be received counselling from healthcare professionals during the process (77.9%). It was found that the most common fears during the pandemic process were the possibility of contracting the virus (68.1%) and dying from the virus (69%) [15]. In this new period, it is necessary to learn how DM patients are affected by the events and to make assessments and take measures to prevent the recurrence of negative consequences in case of a similar situation in the future. In line with this information, this study we conducted to investigate the impact of the pandemic on the clinical condition of patients hospitalised for DF found that the number of patients hospitalised for DF increased during the pandemic period, as did the severity of the wound, duration of admission and amputation requests (increasing from 11.2% to 20%) (*p* < 0.05).

Of all chronic complications of DM, DF problems are the most common cause of hospitalisation [16]. During the pandemic period, bed shortage due to COVID-19 patient admissions, reductions in community health services and anxiety induced by COVID-19 negatively impacted DF patients’ behaviour in seeking regular outpatient visits and treatment adherence. This reportedly led to late hospitalisations and increased hospitalisations for diabetes-related foot complications [17,18]. Ten of the twelve studies analysed in a systematic review showed an increase in emergency admissions and amputations for DF during the pandemic period compared to the pre-pandemic period [19]. In our study, the number of DM patients hospitalised and hospitalisations during the pandemic decreased, while the number of DF patients increased (*p* < 0.001). During the pandemic period, 90% of patients admitted for DM were hospitalised, while the proportion of patients admitted to hospital for DF increased among all DM patients admitted to hospital (*p* < 0.001). These data show that patients with DM- and DF-related problems tended to remain at home to cope with symptoms and do not go to the hospital unless they have to. When admitted to the hospital, patients with very serious conditions required inpatient treatment. These results show once again the importance of maintaining routine controls.

DF is a chronic and late complication of DM and causes a significant decrease in quality of life due to recurrent hospitalisations, disability and profound socioeconomic effects [7,11]. While it was reported that major amputations increased significantly during the pandemic, it was emphasised that this was due to delays in accessing care and advanced disease stages at presentation. However, key risk factors identified, including being over 65 years of age, leukocytosis, sepsis and diabetic polyneuropathy, underline the need for early diagnosis and intervention in DF disease [20]. The first lesson we learned from the pandemic is the need to have a DF care model that is flexible, fluid and adaptable to change. Accordingly, guidelines recommended the use of online resources for patient education, encouraging self-examination of the feet and regular foot care, and telemedical counselling for DF ulcers for the continuation of preventive foot care practises [11,21]. However, studies conducted during the pandemic period show that not all patients can access these practises. In one study, it was found that the time from the onset of symptoms to clinical presentation was significantly longer [31 (1–105) days versus 27 (0–78) days, (*p* = 0.017)], and higher white cell counts (*p* = 0.014) and CRP (*p* = 0.004) levels were found [22]. In our study, although the ESR and CRP values of patients admitted during the pandemic period were higher, there was no significant difference between the periods in metabolic values at the time of hospital admission (*p* > 0.05). However, it was striking that DF patients hospitalised during the pandemic had a longer duration of diabetes. This finding may be explained by the fact that the risk of DF increases with increasing disease duration. Another important finding was that in the pre-pandemic period, patients with DF were in the majority, while during the pandemic period, the majority of patients had no history of DF (*p* < 0.05). It is hypothesised that this finding could be due to the stress caused by the pandemic and changes in daily life (e.g., diet, exercise and sport).

Lower limb amputations that occur as a result of poor diabetes management are life-threatening, debilitating and costly complications that lead to a short life expectancy. Diabetes-related lower limb amputations lead to re-amputation after the first lower limb amputation. However, the risk can be significantly reduced through better care and the promotion of healthy habits in patients with diabetes [23,24]. To reduce the risk through proper promotion and care, it is recommended that all DM patients, but especially those at high risk for DF, should see a diabetes and foot specialist every three months [25,26]. However, it is known that DM patients were unable to benefit from routine care during the pandemic period, and there was a significant decrease in foot examinations [8,12,14,27]. Viswanathan and Nachimuthu (2023) [25] found that the number of major amputations increased by 54.1% during the pandemic period (*n* = 37) compared to the pre-pandemic period (*n* = 24). It was emphasised that this increase could be due to irregular/missed hospital visits, poor diet, non-compliance with drug treatment and physical inactivity [25]. Similar to the results of this study, previous studies reporting an increase in amputations during the pandemic period have linked the increase in amputations to the severity of infection and delayed referral [1,28,29,30,31,32]. Some studies examining short-term follow-up outcomes and implementing strategies to reduce early and specific foot complications reported that rates of DF ulcers and related amputations did not change during the pandemic period [18,26,33]. However, there are also studies suggesting that the incidence of DF ulcers decreased in patients whose mobility decreased due to restrictions and prohibitions, and thus the negative consequences also decreased [34,35,36,37]. In the prediction model study using machine learning by Du et al. (2022) [19], it was found that the three most important baseline variables predicting the occurrence of DF amputations during the pandemic period were prehospital time, foot ischaemia and low serum albumin levels [19]. In our study, the advanced stage of DF in hospitalised during the pandemic period, the decreased wound healing rate during the treatment process, and the increased rate of transtibial amputations are due to the longer time from the detection of the wound to hospitalisation.

### 4.1. Strengths and Limitations of Study

This study has some limitations. First, it has a retrospective design. Second, our data are limited to a single centre, which makes it difficult to generalise the results. However, the current study has several strengths. Our data are homogeneous, as all our patients were diagnosed with DM. However, patients diagnosed with COVID-19 during the pandemic period were excluded from this study to ensure homogeneity of the groups.

### 4.2. Implications for Research and/or Practice

When faced with a situation such as a pandemic, the most important thing in the management of DF disease is to provide appropriate treatment to patients safely at home, if possible, but if not possible and severely affected, to provide effective treatment in outpatient facilities and hospitals [7]. But even more important in DF management is the prevention and early recognition of DF ulcers [20]. According to current standards of care, a multidisciplinary approach to annual foot assessment and foot care is essential [24]. For this purpose, it is emphasised that web-based diabetes self-management education and support systems to be provided by a team consisting of family physicians, endocrinologists, diabetes education nurses, nutritionists, ophthalmologists, podiatrists and psychiatrists will play an important role in diabetes care in adverse conditions such as pandemic where face-to-face care is limited [38]. In addition, it should be kept in mind that diabetes management during such times does not involve only blood glucose monitoring, and constant reminders should be given to other diseases and complications associated with diabetes [39]. Diabetic foot ulcer follow-up training should be given to individuals with diabetes who cannot come to the hospital due to the pandemic. However, it has been emphasised that with the help of many recently developed technologies related to DF, from smart socks to smart insoles, smart mats, smart drainage systems and smart thermography imaging systems, risk factors can be monitored, and early intervention and treatment can be provided to reduce adverse outcomes [3].

## 5. Conclusions

During the COVID-19 pandemic, there were interruptions in the regular check-ups of people with chronic diseases such as diabetes. During these checks, the regular diabetes nurse training sessions for patients could not be carried out. As a result, opportunities for assessment, control and early treatment of risk factors for DF diminished. Like the rest of the world, our country’s healthcare system was unprepared for the pandemic. In our study, we found that the number of patients hospitalised for DF increased during the pandemic period, as did the severity of the wound, length of admission and radical treatment interventions. These results are considered very important, as they show the indirect impact of the COVID-19 pandemic on people with diabetes and DF. They also show the importance of easy and routine access to foot care when it comes to managing wounds and preventing amputations. Healthcare systems need to prepare for the treatment of DF to avoid similar outcomes in the event of a pandemic. Permanent and sustainable arrangements should be implemented for the prevention and early detection of DF ulcers, such as ongoing patient education, remote monitoring and support systems, home treatment options and the use of smart systems (e.g., socks, mats and thermal imaging) to monitor risk factors. More importantly, it is thought that health professionals who have responsibilities for patient education and follow-up in DF care should be willing and equipped to use time- and space-independent digital environments and smart systems to support patient follow-up and education.

## Figures and Tables

**Table 1:** Comparison of the number of hospital admissions and inpatients by period.

	Pre-Pandemic	Pandemic	*p*
Number of applications DM/DF, *n*	8398/45	2789/80	**<0.001**
Number of hospitalisations DM/DF, *n*	2721/31	2512/43	0.1475
DM hospitalisation, %	32.40	90.00	**<0.001**
DF applications, %	0.53	2.86	**<0.001**
DF hospitalisation among applications with DM, %	0.36	1.54	**<0.001**
DF hospitalisation among hospitalised with DM, %	1.13	1.71	0.1475
DF hospitalisation among DF patients, %	68.88	53.75	0.1433

DM: diabetes mellitus and DF: diabetic foot.

**Table 2:** Distribution of sociodemographic data.

		Pre-Pandemic *n* %		Pandemic *n* %		Statistical Analysis
Age	*years (mean ± SD)*	59.35 ± 10.33		60.92 ± 13.10		t = −0.691 *p* = 0.151
Gender	Female	14	31.1	27	33.8	χ^2^ = 0.091
	Male	31	68.9	53	66.2	*p* = 0.462
Marital status	Married	38	84.4	66	82.5	χ^2^ = 0.078 *p* = 0.494
	Single	7	16.6	14	17.5	
Place of residence	Province	21	46.7	34	42.4	χ^2^ = 0.296 *p* = 0.862
	District	21	46.7	39	48.8	
	Village	3	6.6	7	8.8	
Smoking	Yes	6	13.3	11	13.8	χ^2^ = 0.004 *p* = 0.589
	No	39	86.7	69	86.2	
Alcohol use	Yes	4	8.9	4	5.0	χ^2^ = 0.727 *p* = 0.311
	No	41	91.1	76	95.0	

t: Student’s *t*-test and χ^2^: chi-square.

**Table 3:** Disease characteristics.

		Pre-Pandemic *n* %		Pandemic *n* %		Statistical Analysis
Diabetes duration	*years (mean ± SD)*	15 (1–30)		20 (1–40)		MWU = 1346.500 ***p* = 0.019**
Follow-up status	Regular	16	35.6	41	51.2	χ^2^ = 2.860 *p* = 0.066
	Irregular	29	64.4	39	48.8	
DF history	Yes	26	57.8	32	40.0	χ^2^ = 3.660 ***p* = 0.042**
	No	19	42.2	48	60.0	
Treatment	NIAD	10	22.2	12	15.0	χ^2^ = 1.236 *p* = 0.539
	Insulin	20	44.4	42	52.5	
	NIAD + Insulin	15	33.4	26	32.5	
Concomitant disease	Yes	7	15.6	14	17.5	χ^2^ = 0.078 *p* = 0.494
	No	38	84.4	66	82.5	
Concomitant finding	Yes	2	4.4	12	15.0	χ^2^ = 3.226 *p* = 0.061
	No	43	95.6	68	85.0	

NIAD: non-insulin antidiabetic, MWU: Mann–Whitney U test and χ^2^: chi-square.

**Table 4:** Metabolic characteristics.

	Pre-Pandemic Median (Min–Max)	Pandemic Median (Min–Max)	Statistical Analysis
Hg, *mean ± SD*	11.53 ± 2.18	11.60 ± 1.96	t = −0.180, *p* = 0.373
FBG, mg/dl	186 (79–452)	171.50 (85–360)	MWU = 1549.000, *p* = 0.197
HgA1c, %	9 (5.3–17.8)	8.7 (5.3–14.3)	MWU = 1567.500, *p* = 0.232
CRP, mg/L	10.8 (0.32–196)	15.25 (2–207)	MWU = 1485.000, *p* = 0.105
ESR, mm/h	34 (12–247)	41.50 (1.42–137)	MWU = 1603.000, *p* = 0.311
Leucocytes, 10^9^/L	9.01 (4.51–25.50)	8.93 (5.29–18.04)	MWU = 1773.000, *p* = 0.890
Creatinine, mg/dL	0.90 (0.61–6.38)	1.00 (0.53–6.44)	MWU = 1479.500, *p* = 0.099

Hg: haemoglobin, FBG: fasting blood glucose, CRP: C-reactive protein, ESR: erythrocyte sedimentation rate, t: Student’s *t*-test and MWU: Mann–Whitney U test.

**Table 5:** Diabetic foot data.

		Pre-Pandemic *n* %		Pandemic *n* %		Statistical Analysis
Time until hospital admission	*days, median (min-max)*	7 (1–30)		10 (1–90)		MWU = 1394.000 ***p* = 0.035**
Wagner Classification	Grade 1	7	15.5	4	5.0	χ^2^ χ^2^ = 12.352 ***p* = 0.015**
	Grade 2	9	17.8	23	27.5	
	Grade 3	15	31.1	36	43.8	
	Grade 4	10	20.0	5	5.0	
	Grade 5	7	15.6	15	18.8	
Osteomyelitis	Yes	19	42.2	39	48.8	χ^2^ = 0.493 *p* = 0.304
	No	26	57.8	41	51.2	
Hospitalisation status	Yes	31	68.9	43	53.8	χ^2^ = 2.733 *p* = 0.710
	No	14	31.1	37	46.3	
Hospitalisation duration	*days, median (min–max)*	14 (2–37)		8 (2–37)		MWU = 504.000 *p* = 0.074
Treatment *	Debridement	12	26.7	23	28.7	*p* > 0.05
	Hyperbaric oxygen	2	4.4	4	5.0	
	Vacuum-assisted wound care	1	2.2	7	8.8	
	Graft	3	6.7	3	3.8	
Result of applied treatment	Wound healing	28	62.2	24	30.0	χ^2^ = 14.398 ***p* = 0.002**
	Wound regression	6	13.3	30	37.5	
	Finger amputation	6	13.3	10	12.5	
	Below knee amputation	5	11.2	16	20.0	

MWU: Mann–Whitney U test and χ^2^: chi-square. * More than one option is marked. Therefore, each treatment group (yes/no) was evaluated within itself with the chi-square test.

## Data Availability

The data of the patients’ medical history will be provided by the authors without hesitation if requested.

## Abbreviations

The following abbreviations are used in this manuscript:

DF	Diabetic Foot
DM	Diabetes Mellitus
WHO	World Health Organisation
CRP	C Reactive Protein
ESR	Erythrocyte Sedimentation Rate
Hg	Haemoglobin
FBG	Fasting Blood Glucose

## References

[1] Carro G.V., Carlucci E.M., Torterola I., Breppe P., Ticona Ortiz M.Á., Palomino Pallarez J.E. (2020). Diabetic foot and COVID-19. Medical consultation and severity of lesions compared to 2019. Medicina (B Aires).

[2] Ministry of Health of the Republic of Türkiye. https://covid19.saglik.gov.tr/TR-66494/pandemi.html.

[3] Najafi B. (2020). Post the pandemic: How will COVID-19 transform diabetic foot disease management. J. Diabetes. Sci. Technol..

[4] Kutlutürk F. (2020). The COVID-19 Pandemic and Diabetes Mellitus/COVID-19 Pandemisi ve Diabetes Mellitus. Turk. J. Diab. Obes..

[5] World Health Organization. https://www.who.int/westernpacific/emergencies/covid-19/information/high-risk-groups.

[6] Lazzarini P.A., Cramb S.M., Golledge J., Morton J.I., Magliano D.J., Van Netten J.J. (2023). Global trends in the incidence of hospital admissions for diabetes-related foot disease and amputations: A review of national rates in the 21st century. Diabetologia.

[7] Boulton A.J.M. (2021). Diabetic foot disease during the COVID-19 Pandemic. Medicina.

[8] Pušnik L., Gabor A., Radochová B., Janáček J., Saudek F., Alibegović A., Serša I., Cvetko E., Umek N., Snoj Ž. (2025). High-Field Diffusion Tensor Imaging of Median, Tibial, and Sural Nerves in Type 2 Diabetes With Morphometric Analysis. J. Neuroimaging.

[9] Miranda C., Zanette G., Da Ros R. (2022). Diabetic foot disease during the COVID-19 pandemic: Lessons learned for our future. Arch. Med. Sci. Atheroscler. Dis..

[10] Vukas H., Piljic D., Kadić-Vukas S., Piljic D. (2021). Challenges in diabetic foot treatment during pandemic of COVID-19. Saudi. Med. J..

[11] Ergişi Y., Özdemir E., Altun O., Tıkman M., Korkmazer S., Yalçın M.N. (2022). Indirect impact of the COVID-19 pandemic on diabetes-related lower extremity amputations: A regional study. Jt. Dis. Relat. Surg..

[12] Saraçoğlu E., Aydın Avcı İ. (2021). Determination of the diabetes patients concerns and care needs about the COVID-19 pandemic/Diyabet hastalarının COVID-19 salgınıyla ilgili endişelerinin ve bakım ihtiyaçlarının belirlenmesi. Turk. J. Diab. Obes..

[13] Shin L., Bowling F.L., Armstrong D.G., Boulton A.J.M. (2020). Saving the diabetic foot during the COVID-19 pandemic: A tale of two cities. Diabetes Care.

[14] Gong J.Y., Collins L., Barmanray R.D., Pang N.S.K., Le M.V., Wraight P.R. (2024). The experience of an adult diabetic foot unit continuing face-to-face consults during the COVID-19 pandemic. Intern. Med. J..

[15] Rubin G., Feldman G., Dimri I., Shapiro A., Rozen N. (2023). Effects of the COVID-19 pandemic on the outcome and mortality of patients with diabetic foot ulcer. Int. Wound. J..

[16] Flynn S., Kirwan E., MacGilchrist C., McIntosh C. (2024). The impact of COVID-19 on the care of diabetic foot ulcers: A scoping review. J. Tissue Viability..

[17] Jaly I., Iyengar K., Bahl S., Hughes T., Vaishya R. (2020). Redefining diabetic foot disease management service during COVID-19 pandemic. Diabetes. Metab. Syndr..

[18] Goyal G., Majumdar S., Biswas-Bose U., Shrivastava M.R., Mukherjee J.J., Banka S.P., Kapoor S., Jude E. (2024). The effect of the different waves of COVID-19 pandemic on the outcome of diabetic foot ulcers. Int. J. Low. Extrem. Wounds..

[19] Du C., Li Y., Xie P., Zhang X., Deng B., Wang G., Hu Y., Wang M., Deng W., Armstrong D.G. (2022). The amputation and mortality of inpatients with diabetic foot ulceration in the COVID-19 pandemic and postpandemic era: A machine learning study. Int. Wound. J..

[20] Rusu E., Catrina E.L., Brezean I., Georgescu A.M., Vișinescu A., Georgescu D.A.V., Mioara C.A., Dobra G.M., Verde I., Stanciu S. (2024). Lower extremity amputations among patients with diabetes mellitus: A five-year analysis in a clinical hospital in Bucharest, Romania. Medicina.

[21] Nachimuthu S., Ahmed-Khan B., Viswanathan V. (2023). Managing diabetic foot complications during COVID-19 lockdown in India: A survey. Int. J. Low. Extrem. Wounds..

[22] Zayed H., Musajee M., Thulasidasan N., Sayed M., Francia F., Green M., Arissol M., Lakhani A., Biasi L., Patel S. (2022). Impact of COVID-19 pandemic on the outcomes in patients with critical limb threatening ischaemia and diabetic foot infection. Ann. Surg..

[23] Ezzatvar Y., García-Hermoso A. (2023). Global estimates of diabetes-related amputations incidence in 2010–2020: A systematic review and meta-analysis. Diabetes. Res. Clin. Pract..

[24] Rosien L., van Dijk P.R., Oskam J., Pierie M.E.N., Groenier K.H., Gans R.O.B., Bilo H.J.G. (2023). Lower extremity amputation rates in people with diabetes mellitus: A retrospective population based cohort study in Zwolle Region, the Netherlands. Eur. J. Vasc. Endovasc. Surg..

[25] Viswanathan V., Nachimuthu S. (2023). Major lower-limb amputation during the COVID pandemic in South India. Int. J. Low. Extrem. Wounds..

[26] Rastogi A., Hiteshi P., Bhansali A.A., Jude E.B. (2021). Virtual triage and outcomes of diabetic foot complications during COVID-19 pandemic: A retro-prospective, observational cohort study. PLoS ONE.

[27] AlMajali A.S., Richards T., Yusuf S.W., Telgenkamp B. (2024). Vascular service provision during the COVID-19 pandemic worsened major amputation rates in socially deprived diabetic populations. Front. Endocrinol..

[28] Caruso P., Longo M., Signoriello S., Gicchino M., Maiorino M.I., Bellastella G., Chiodini P., Giugliano D., Esposito K. (2020). Diabetic foot problems during the COVID-19 pandemic in a tertiary care center: The emergency among the emergencies. Diabetes Care.

[29] Liu C., You J., Zhu W., Chen Y., Li S., Zhu Y., Ji S., Wang Y., Li H., Li L. (2020). The COVID-19 outbreak negatively affects the delivery of care for patients with diabetic foot ulcers. Diabetes Care.

[30] Casciato D.J., Yancovitz S., Thompson J., Anderson S., Bischoff A., Ayres S., Barron I. (2020). Diabetes-related major and minor amputation risk increased during the COVID-19 pandemic. J. Am. Podiatr. Med. Assoc..

[31] Yunir E., Tarigan T.J.E., Iswati E., Sarumpaet A., Christabel E.V., Widiyanti D., Wisnu W., Purnamasari D., Kurniawan F., Rosana M. (2022). Characteristics of diabetic foot ulcer patients pre- and during COVID-19 pandemic: Lessons learnt from a national referral hospital in Indonesia. J. Prim. Care. Community Health.

[32] Schmidt B.M., Munson M.E., Rothenberg G.M., Holmes C.M., Pop-Busui R. (2020). Strategies to reduce severe diabetic foot infections and complications during epidemics (STRIDE). J. Diabetes Complicat..

[33] Mariet A.S., Benzenine E., Bouillet B., Vergès B., Quantin C., Petit J.M. (2021). Impact of the COVID-19 epidemic on hospitalization for diabetic foot ulcers during lockdown: A French nationwide population-based study. Diabet. Med..

[34] de Mestral C., Gomez D., Wilton A.S., Lee D.S., Albalawi Z., Austin P.C., Jacob-Brassard J., Urbach D.R., Al-Omran M., Baxter N.N. (2022). A population-based analysis of diabetes-related care measures, foot complications, and amputation during the COVID-19 pandemic in Ontario, Canada. JAMA Netw. Open.

[35] Lipscomb D., Smith A.S., Adamson S., Rezazadeh E.M. (2020). Diabetic foot ulceration in COVID-19 lockdown: Cause for concern or unexpected benefit?. Diabet. Med..

[36] Valabhji J., Barron E., Vamos E.P., Dhatariya K., Game F., Kar P., Weaver A., Verma S., Young B., Khunti K. (2021). Temporal trends in lower-limb major and minor amputation and revascularization procedures in people with diabetes in England during the COVID-19 pandemic. Diabetes Care.

[37] Kleibert M., Mrozikiewicz-Rakowska B., Bąk P.M., Bałut D., Zieliński J., Czupryniak L. (2022). Breakdown of diabetic foot ulcer care during the first year of the pandemic in Poland: A retrospective national cohort study. Int. J. Environ. Res. Public Health.

[38] Banerjee M., Chakraborty S., Pal R. (2020). Diabetes self-management amid COVID-19 pandemic. Diabetes Metab. Syndr..

[39] Polat G., Ünsal-Avdal E. (2021). Diabetes management and responsibilities of diabetes nurses in covid-19 pandemic/Covid-19 Pandemisinde Diyabet Yönetimi ve Diyabet Hemşirelerinin Sorumlulukları. Başkent Üniversitesi Sağlık Bilimleri Fakültesi Dergisi.

